# Clinical Spectrum of Thyroid Eye Disease in a Tertiary Referral Centre in Eastern India: A Case Series

**DOI:** 10.7759/cureus.117497

**Published:** 2026-10-05

**Authors:** Renukha J, Divya Kesarwani, Bhawesh C Saha, Amit Raj, Dev Kant

**Affiliations:** 1 Department of Ophthalmology, All India Institute of Medical Sciences, Patna, Patna, IND

**Keywords:** compressive optic neuropathy, graves orbitopathy, intravenous corticosteroids, orbital inflammation, thyroid-associated orbitopathy, thyroid eye disease

## Abstract

Thyroid eye disease (TED) is a chronic autoimmune inflammatory disorder characterized by a wide spectrum of clinical manifestations ranging from mild features such as eyelid retraction and periorbital edema to vision-threatening complications like compressive optic neuropathy. The purpose of this case series is to highlight the clinical challenges, therapeutic dilemmas, and management complexities encountered in patients with TED. This case series includes five patients diagnosed with thyroid-associated orbitopathy. Four of five patients presented with proptosis, four had restrictive extraocular myopathy, one presented with isolated upper eyelid retraction, and four had elevated intraocular pressure. Three patients required intravenous methylprednisolone therapy for active disease, whereas two were managed conservatively with ocular lubricants and topical antiglaucoma medications. Disease activity was assessed using the Clinical Activity Score (CAS). Intravenous methylprednisolone was administered in indicated cases if disease was found to be active, moderate to severe, and sight-threatening, as per the European Group on Graves' Orbitopathy (EUGOGO) regimen. Post-treatment, patients demonstrated improvement in visual acuity, reduction in proptosis, and decreased eyelid edema. However, residual ocular deviation and extraocular motility restriction persisted, with minimal improvement. All patients underwent regular three-monthly follow-up for monitoring and rehabilitation. We found that systemic corticosteroid therapy was effective in controlling inflammatory activity and improving vision. However, restrictive myopathy and strabismus showed limited reversibility, indicating progression to the fibrotic phase in some cases. Thus, this case series underscores the chronic and potentially progressive nature of TED and highlights the need for a multidisciplinary, individualized treatment approach. Early recognition and timely intervention are critical in preventing irreversible vision loss. The persistence of functional and cosmetic sequelae necessitates long-term follow-up and, in many cases, staged reconstructive surgeries, thereby contributing to the overall social and psychological burden of the disease.

## Introduction

Thyroid eye disease (TED) is a chronic autoimmune inflammatory disorder involving the orbit and represents the most common extra-thyroidal manifestation of autoimmune thyroid disease. The reported annual incidence of TED is approximately 16 cases per 100,000 population in women and 2.9 cases per 100,000 in men, with a clear female preponderance [1,2].

The estimated prevalence in the general population ranges from 90 to 155 per 100,000 individuals, with an overall prevalence of approximately 0.25% [2]. Among patients with Graves’ disease, nearly 25-50% develop some form of orbital involvement, although the majority (about 77%) have mild disease, while only 1% develop sight-threatening complications such as optic neuropathy [3].

Most commonly, it occurs in association with Graves' disease, although it may also develop in euthyroid or hypothyroid individuals with autoimmune thyroid disease. The disease results from a complex autoimmune process in which thyroid-stimulating hormone receptor and insulin-like growth factor-1 receptor autoantibodies activate orbital fibroblasts, leading to inflammatory cell infiltration, excessive glycosaminoglycan deposition, adipogenesis, and enlargement of the extraocular muscles. These pathological changes increase orbital volume and manifest clinically as eyelid retraction, proptosis, conjunctival congestion, restrictive extraocular myopathy with diplopia, exposure keratopathy and, in severe cases, compressive dysthyroid optic neuropathy (DON) [4,5]. The disease follows a biphasic course consisting of an active inflammatory phase and a subsequent inactive fibrotic phase [6].

Disease activity is commonly assessed using the Clinical Activity Score (CAS), whereas severity-based management is guided by the European Group on Graves' Orbitopathy (EUGOGO) recommendations [7]. Treatment depends on disease activity and severity and includes supportive ocular surface therapy, smoking cessation, restoration of euthyroidism, systemic corticosteroids for active moderate-to-severe and sight-threatening disease with DON, with different dosing recommendations as per EUGOGO, orbital radiotherapy in selected patients, biologic agents such as teprotumumab where available, and rehabilitative procedures including orbital decompression, strabismus surgery, and eyelid correction during the inactive phase.

Despite advances in understanding its pathophysiology, management remains challenging due to variability in disease activity, severity, and treatment response. Therefore, our case series describes the spectrum of clinical presentation, management strategies, and short-term outcomes of five patients with TED managed at a tertiary ophthalmic center.

## Case presentation

This retrospective case series included patients diagnosed with TED who were managed in the Department of Ophthalmology at a tertiary referral center in eastern India, between June 2025 and January 2026. Cases were identified through departmental medical records. Five patients diagnosed with thyroid-associated orbitopathy based on clinical findings and systemic evaluation were studied to illustrate the spectrum of disease presentation, disease activity, associated ocular hypertension, management approaches, and treatment response. Demographic characteristics, thyroid status, presenting symptoms, clinical examination, relevant imaging, treatment, and follow-up outcomes were reviewed from available records. Four of five patients presented with proptosis, four had restrictive extraocular myopathy, one presented with isolated upper eyelid retraction, and four had elevated IOP. Three patients required intravenous methylprednisolone therapy, whereas two were managed conservatively with ocular lubricants and topical antiglaucoma medications (Figures 1-6).

**Figure 1:**
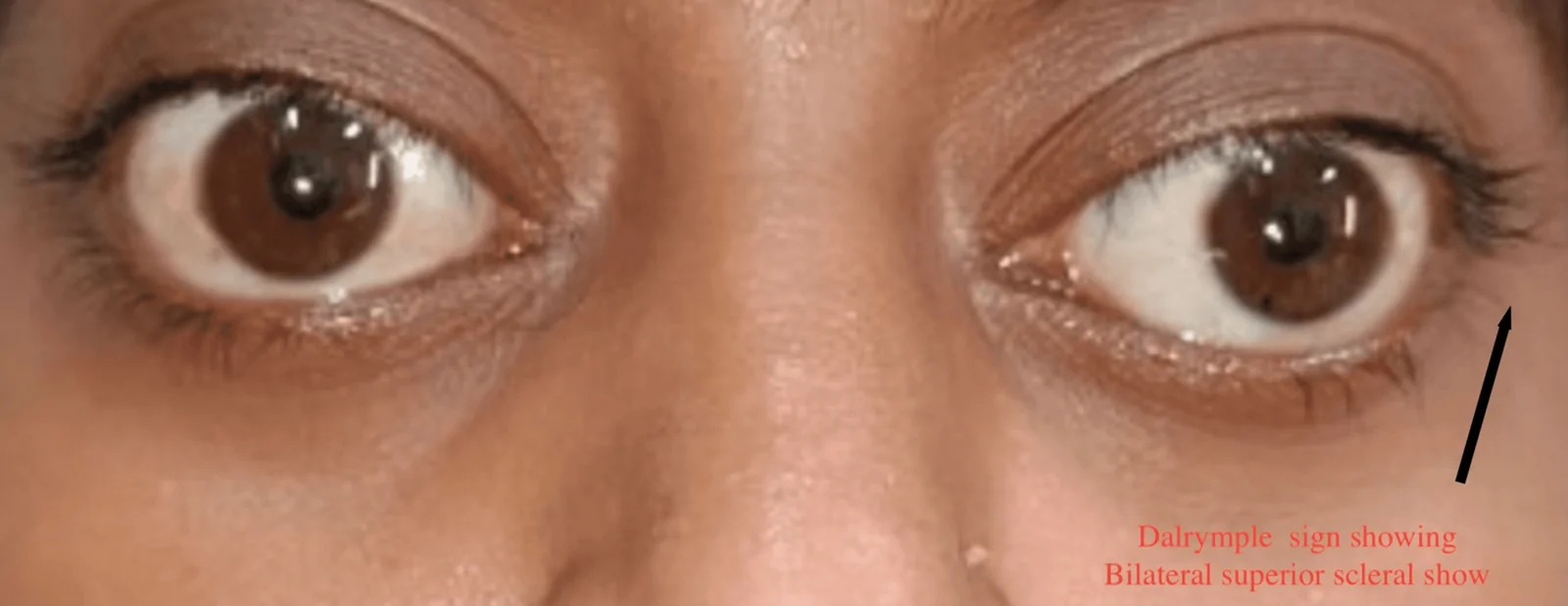
Case 1 showing bilateral proptosis with eyelid retraction. Mild periorbital edema was seen. Dalrymple sign present, showing widening of palpebral fissure height with superior scleral show, representing active thyroid orbitopathy.

**Figure 2:**
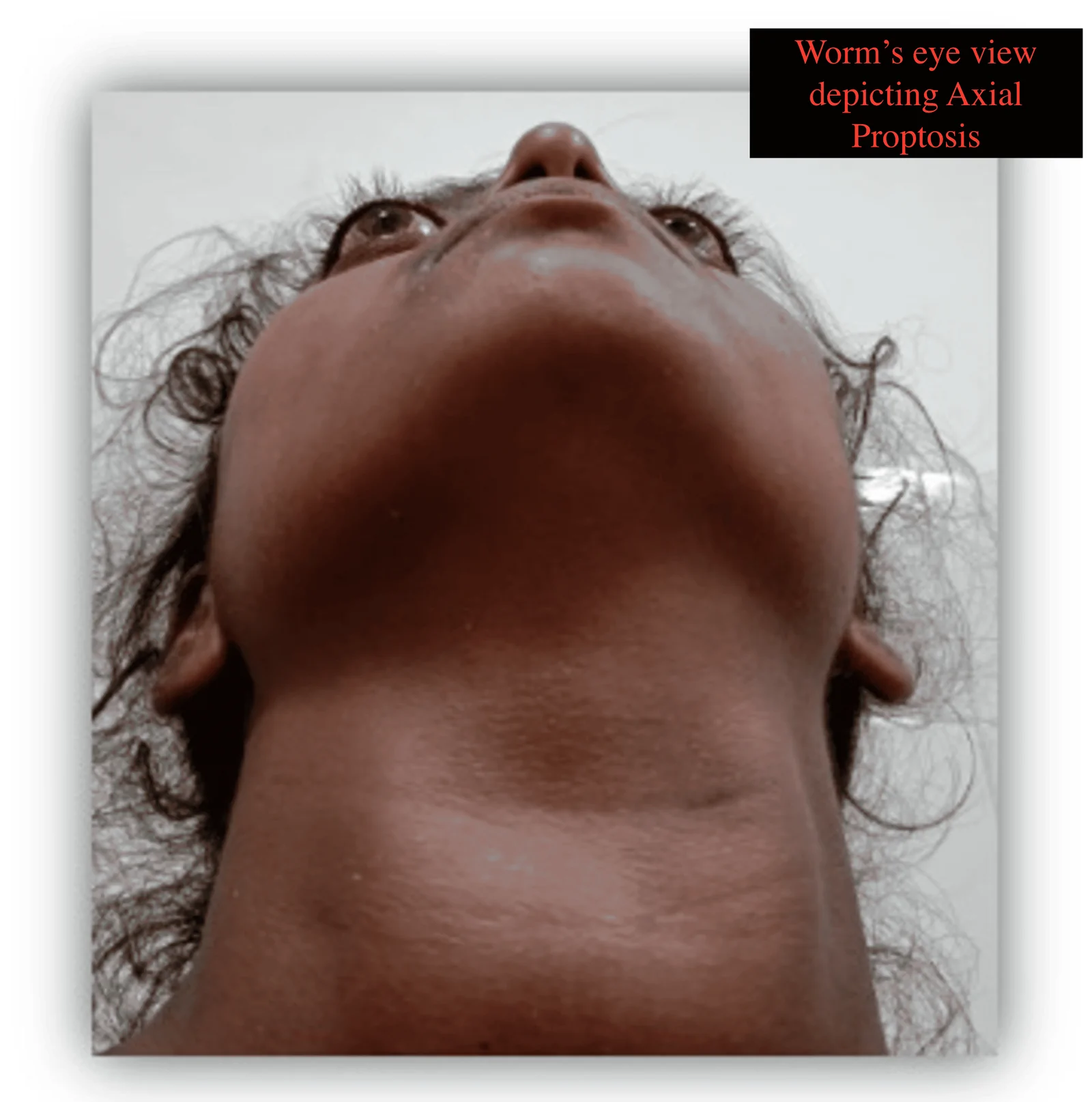
Case 1 showing the clinical presentation of thyroid orbitopathy with worm's eye view representing axial proptosis of both eyes. The worm’s-eye view highlights bilateral axial proptosis with anterior displacement of the globes, a characteristic feature of thyroid-associated orbitopathy. A diffuse enlargement of the thyroid gland (goiter) is also seen in the anterior neck, consistent with underlying Graves’ disease. Written informed consent was obtained from the patient.

**Figure 3:**
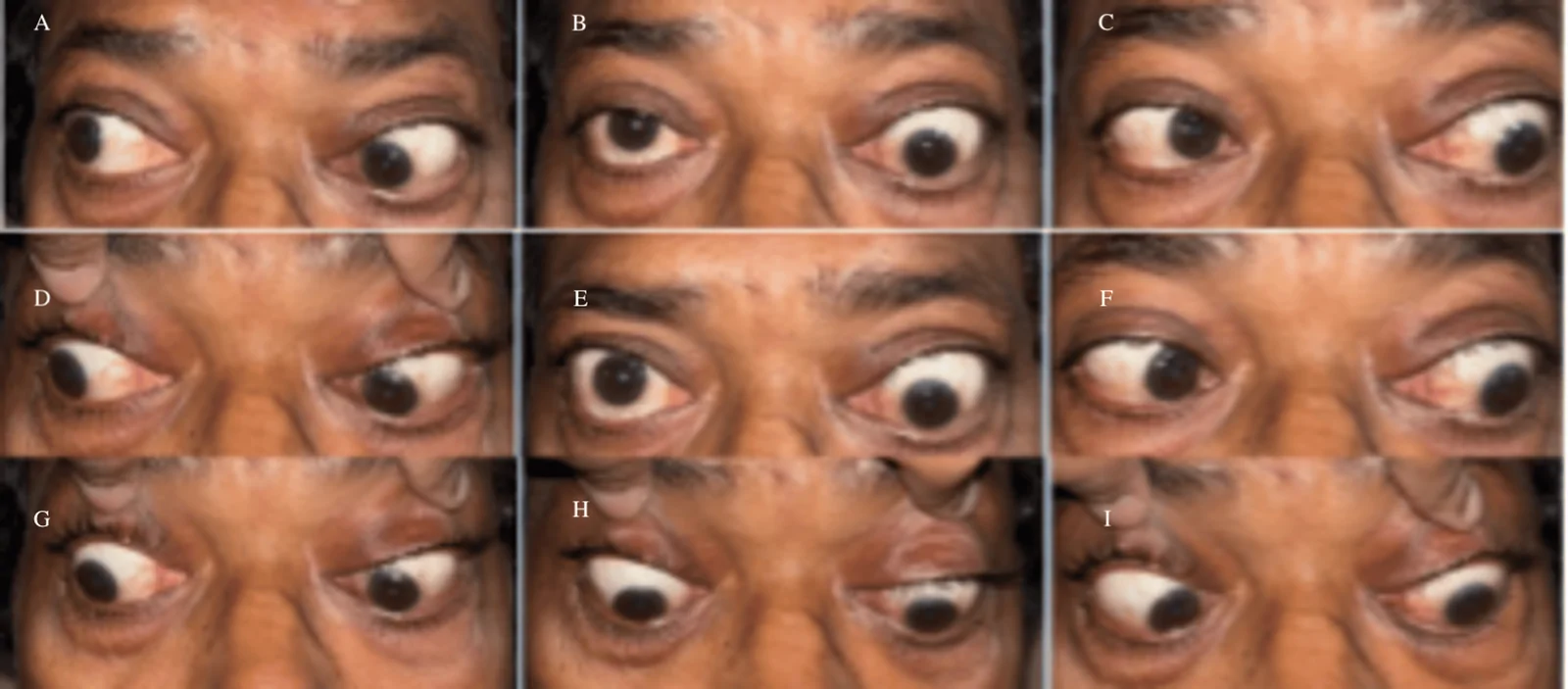
Case 3 showing restricted extraocular motility in all gazes with asymmetric exophthalmos. Nine cardinal positions of gaze demonstrating marked bilateral proptosis with asymmetric limitation of extraocular movements. Restriction is more in elevation and horizontal gaze, consistent with restrictive extraocular muscle involvement secondary to thyroid-associated orbitopathy. Associated lower eyelid retraction, inferior scleral show, and conjunctival congestion are also present. The findings are characteristic of active thyroid eye disease with extraocular muscle dysfunction.

**Figure 4:**
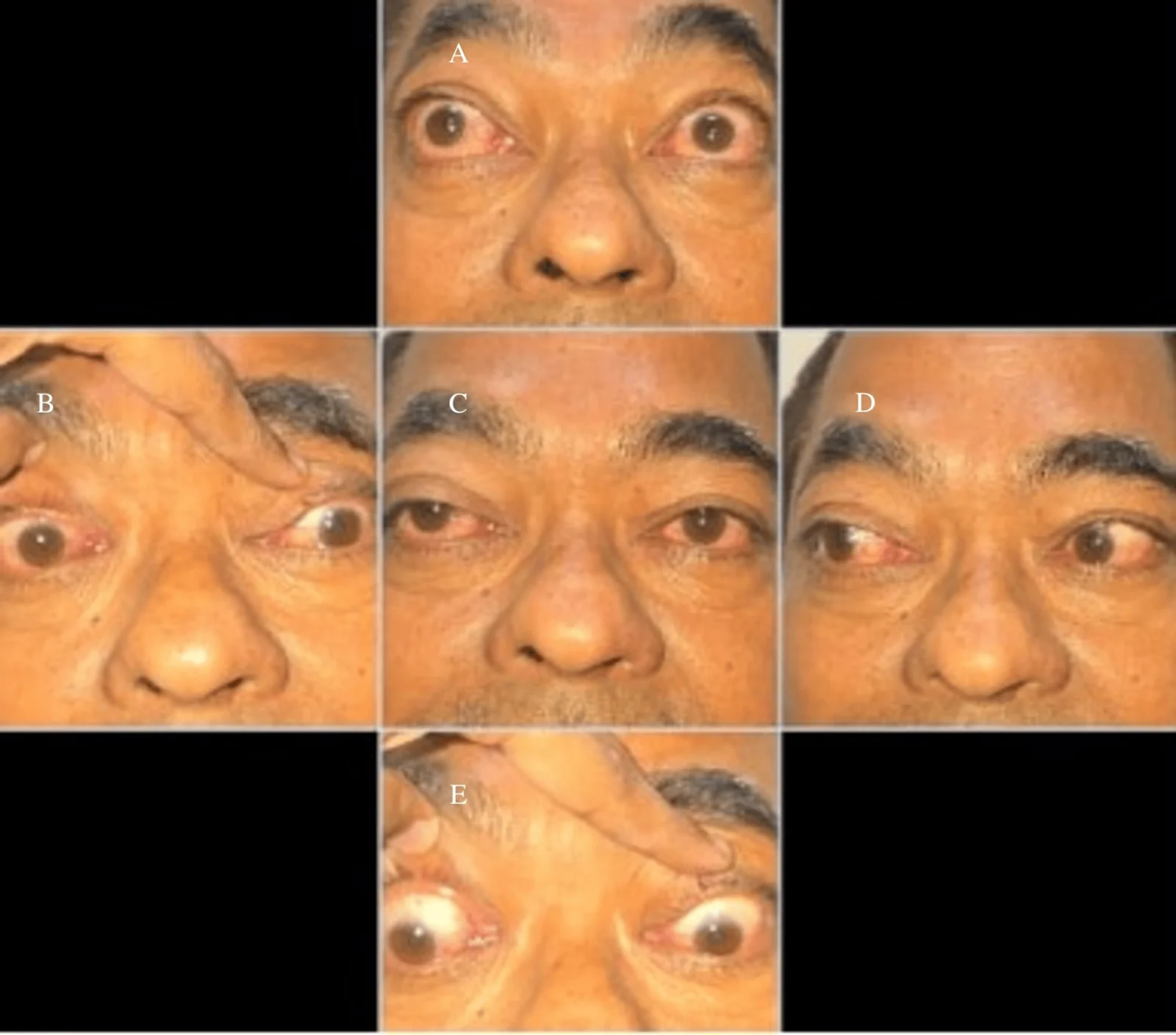
Case 3 post IV methylprednisolone showing partial improvement in clinical appearance of proptosis, periocular congestion and extraocular movements. Post-treatment photographs demonstrating reduction in conjunctival congestion, chemosis, and periocular inflammation. Bilateral axial proptosis and restrictive extraocular motility deficits persist, reflecting the chronic fibrotic component of thyroid eye disease despite resolution of active inflammation. Written informed consent was obtained from the patient.

**Figure 5:**
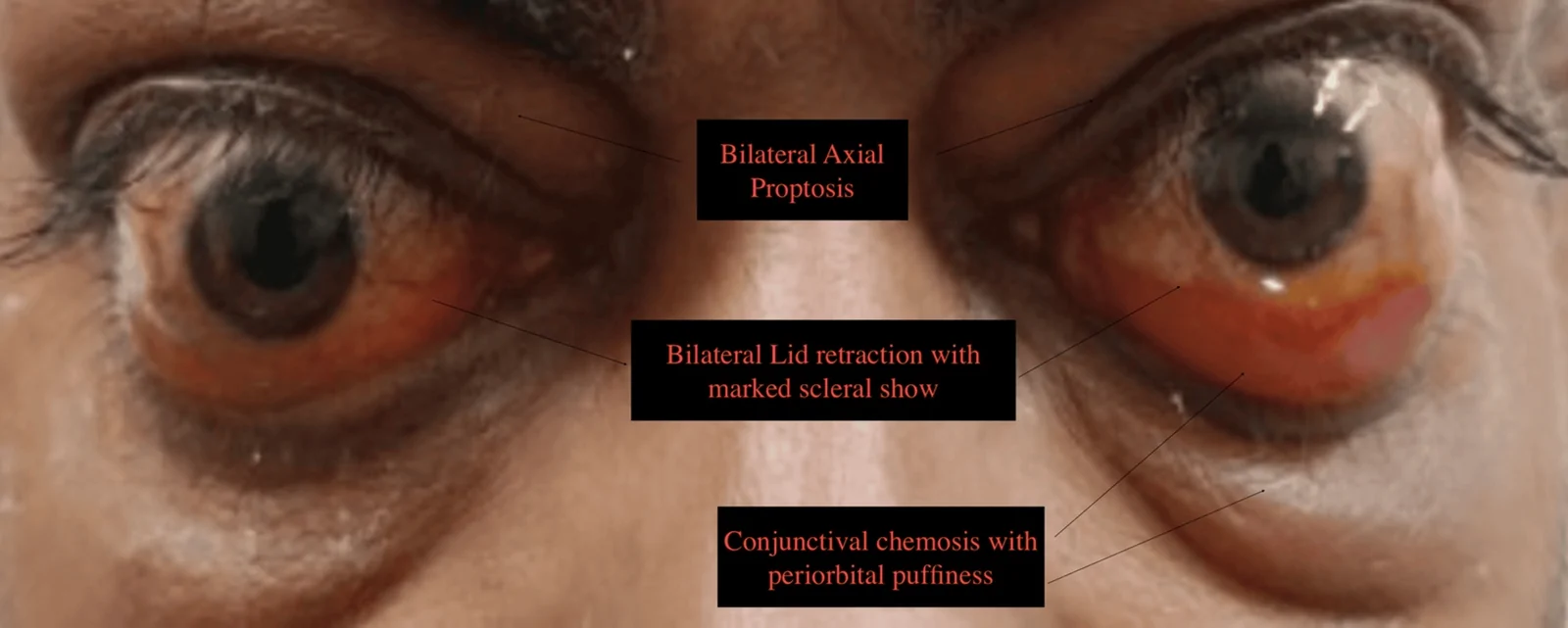
Case 4 with Graves' orbitopathy showing severe bilateral axial proptosis, significant eyelid retraction and conjunctival hyperemia and chemosis with risk of exposure keratopathy. The photograph shows bilateral axial proptosis with marked lower eyelid retraction and inferior scleral show. Conjunctival congestion and chemosis are evident, particularly in the left eye, reflecting active orbital inflammation. These findings are characteristic of active Graves' orbitopathy with significant soft-tissue involvement.

**Figure 6:**
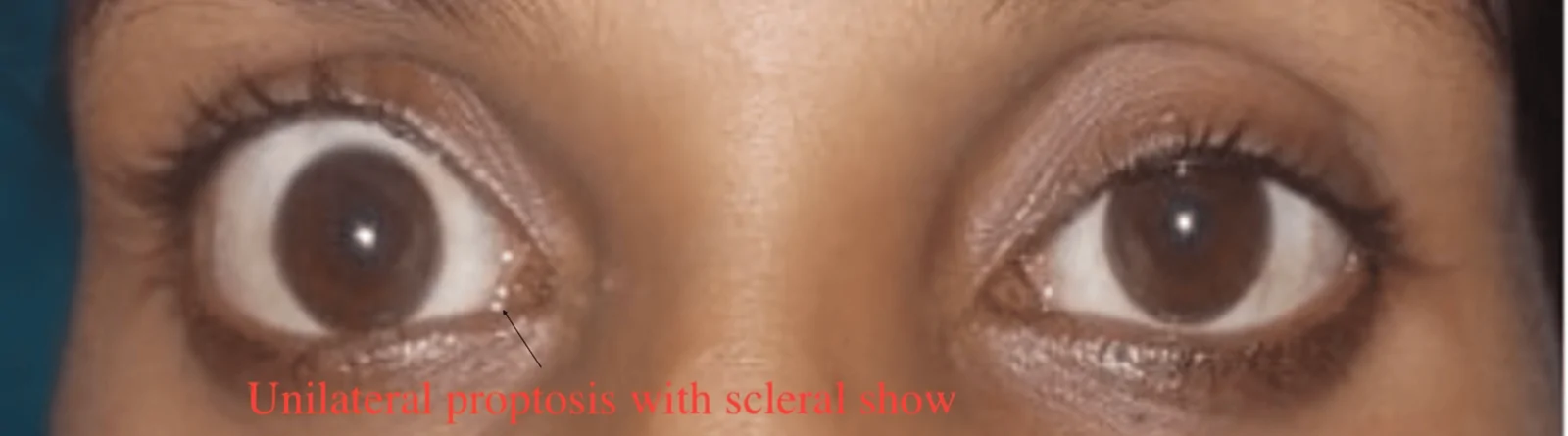
Case 5 showing unilateral right eye proptosis with mild upper eyelid retraction and scleral show. There are no inflammatory signs, suggestive of mild, inactive thyroid-associated orbitopathy.

All patients underwent best-corrected visual acuity (BCVA) by Snellen chart, IOP measurement, extraocular motility assessment, and anterior and posterior segment evaluation. The diagnosis of TED was established based on compatible clinical findings, including eyelid retraction, proptosis, restrictive extraocular muscle dysfunction, conjunctival inflammation, and elevated IOP where present, together with thyroid function assessment and endocrinology evaluation. Disease activity was graded using the CAS (Table 1) [8].

**Table 1:** Clinical activity score for TED. One point is given for each parameter assessed. The sum of all points defines the Clinical Activity Score (CAS). Active TAO if CAS > 3/7 at initial presentation and CAS > 4/10 in successive examinations.

For initial CAS score items 1-7		
Pain	1	Spontaneous orbital pain
	2	Pain on eye movement (in the last four weeks)
Redness	3	Eyelid erythema
	4	Conjunctival hyperemia (>1 quadrant)
Swelling	5	Eyelid swelling
	6	Conjunctiva chemosis
	7	Caruncle inflammation
Follow up after 1-3 months score items including 8-10		
Impaired function	8	Increase in proptosis >2mm.
	9	Decrease in motility in any direction >5 deg
	10	Decrease in visual acuity ≥ 1 line on Snellen.

Because of retrospective design and the small sample size, certain objective measurements, including Hertel exophthalmometry, prism deviation measurements, and thyroid autoantibody profiles, were not available for every patient. Similarly, orbital imaging was performed in selected patients when clinically indicated to confirm extraocular muscle enlargement and exclude alternative orbital pathology.

Case 1 was a 20-year-old woman with hyperthyroidism and a goiter presented with bilateral lid edema, retraction with superior scleral show depicting Dalrymple sign and axial proptosis, clinically seen at worm's eye view. Her BCVA was 6/6 OU and IOP was elevated to 24 and 26 mmHg respectively. Extraocular movement (EOM) was restricted in all gazes with left eye subtle vertical misalignment with hypotropia. There was no diplopia. Fundus showed CDR 0.4 OU with normal neuroretinal rim (NRR). Proptosis was measured with Hertel’s exophthalometer, base diameter as 110 and readings as 29 and 30 OU. Her CAS was 3/7, indicating mild active TED. The patient was managed conservatively with carboxymethylcellulose 1% eye drops six times daily, hydroxypropyl methylcellulose ointment at bedtime, and topical brimonidine-timolol combination twice daily for ocular hypertension. The patient remained stable on follow-up at three months without progression of disease and was maintained under observation. BCVA remained 6/6, IOP reduced to 19 and 20 mmHg, respectively, Proptosis improved but EOM restriction persisted. CAS improved to 2/7 (Figures 1, 2).

Case 2 was a 34-year-old male, smoker with hyperthyroidism and a history of antitubercular therapy presented with bilateral proptosis, orbital pain, diminished vision and marked restriction of EOMs. Diplopia was present. His BCVA was 6/36 OD and 6/24 OS, IOP was 32 and 28 mmHg respectively. On examination, bilateral eyelid retraction, conjunctival chemosis and congestion were present, pupil was sluggish reacting, RAPD absent, and color vision abnormal. Fundus showed hyperemic disc with CDR 0.5, venous tortuosity and few choroidal folds at macula. The disease was clinically active, and the CAS was 4/7. Owing to the severity of inflammation and visual compromise, 1gm intravenous methylprednisolone pulse therapy for three days was administered for three consecutive days then at one week followed by oral tapering dose of steroids for 12 weeks was administered along with oral acetazolamide 250 mg thrice daily for a week and topical brimonidine-timolol combination. The patient showed improvement in visual acuity and inflammatory signs, and BCVA improved to 6/18 and 6/9, respectively. IOP was 24 and 22 mmHg. The CAS improved to 2/10. However, residual restrictive ophthalmopathy persisted at follow-up at three months.

Case 3 was a 58-year-old male. He had hyperthyroidism and presented with progressive bilateral protrusion, redness and retro-orbital discomfort. He complained of diplopia and restriction of ocular movements, which gradually worsened with the time. His BCVA was 6/36 OD and 6/24 OS. Anterior segment examination revealed a brisk pupillary reaction without evidence of a relative afferent pupillary defect, and lens was cataractous with OU nuclear sclerosis grade 2. External examination revealed bilateral axial proptosis (OD>OS) with upper eyelid retraction with marked scleral show, conjunctival congestion, and exposure-related ocular surface changes. Extraocular motility assessment showed significant restriction in multiple gazes, particularly involving elevation and horizontal movements, consistent with restrictive thyroid-associated orbitopathy. The CAS was 3/7, with spontaneous retro-orbital pain, pain on attempted eye movements, and conjunctival congestion. IOP was elevated in primary gaze i.e. 35 and 29 mmHg respectively. Fundus showed healthy optic discs with CDR 0.5 OU, normal NRR. Visual field testing showed generalized depression (may be explained due to cataract). There was no evidence of compressive optic neuropathy based on presenting visual acuity, preserved color vision, absence of RAPD, normal disc appearance and imaging findings. Magnetic resonance imaging of the orbit demonstrated bilateral enlargement of the extraocular muscles, predominantly involving the medial and inferior recti, with relative sparing of the tendinous insertions, confirming the diagnosis of TED. Hertel exophthalmometry revealed bilateral proptosis with the measurements of 30 and 29 mm (at a base diameter of 124). The patient was treated with intravenous methylprednisolone 500 mg weekly for six weeks followed by 250mg IV weekly for six weeks (cumulative dose of 4.5 g over 12 weeks) as per EUGOGO guidelines for active moderate-severe disease. Systemic and topical antiglaucoma medications for ocular hypertension were added. At the three-month follow-up, orbital pain and conjunctival inflammation improved significantly, with partial reduction in proptosis. Vision improved, BCVA 6/9 OU, CAS improved to 2/7, and IOP reduced to 28 & 22 mmHg, respectively. Proptosis reduced; however, residual extraocular muscle restriction persisted despite treatment (Figures 3, 4).

Case 4 was a 42-year-old male with hyperthyroidism presented with pain, redness and double vision with forward protrusion of both eyes for six months. His BCVA was 6/18 OD and 6/9 OS, and IOP 41 and 35 mmHg on GAT, respectively. On examination, bilateral proptosis and lid retraction with inferior scleral show were seen. Anterior segment showed OU conjunctiva congestion with prominent temporal vessels, clear cornea, ACD deep and quiet, pupil reaction normal, RAPD was absent. Fundus was CDR 0.5 and 0.4, hyperemic NRR, respectively. Color vision and contrast testing was found to be abnormal in both the eyes. Proptosis evaluation and measurements were done. Baseline readings were 35 and 34 mm in both eyes at a base diameter of 120. EOM was markedly restricted in both eyes in all gazes. Diplopia charting was done. The CAS was 4/7. Contrast-enhanced MRI brain and orbit showed enlarged EOM belly on bilateral sides sparing tendinous insertions (involving B/L inferior recti, medial recti and right lateral rectus muscle) with proptosis and orbital apex crowding leading to bilateral optic nerve compression. In view of the reduced visual acuity, bilateral abnormalities of color vision and contrast sensitivity, and radiological evidence of apical crowding with optic nerve compression, the patient was considered to have bilateral sight-threatening TED with DON. Management included intravenous methylprednisolone pulse therapy (1gm IV for three days followed by weekly for two weeks until there were signs of improvement of ophthalmopathy) and then tapering dose of oral steroid for 8-12 weeks was given along with topical Travoprost once daily bed time and combination of brinzolamide plus timolol twice daily. Clinical follow-up at three months demonstrated a reduction in orbital inflammation and proptosis; BCVA improved to 6/9 OD and 6/6 OS, IOP reduced but remained high on AGMS. The CAS improved to 2/10. However, persistent motility restriction remained, suggestive of residual fibrotic extraocular muscle involvement (Figure 5).

Case 5 was a 25-year-old woman with hyperthyroidism presented with unilateral upper eyelid retraction without significant proptosis or visual impairment. BCVA was 6/6 OU, and IOP anterior and posterior segment findings were normal. The CAS was 1/7 and IOP was within normal limits (18 mmHg) OU. She was managed conservatively and remained clinically stable during follow-up at three months (Figure 6).

Three patients received intravenous methylprednisolone; two were treated for active moderate-to-severe TED, while one had clinical features suggestive of sight-threatening disease/DON. Elevated IOP was managed with topical antiglaucoma medications and copious lubricants. Patients were followed periodically to monitor response, progression, and need for further intervention (Table 2).

**Table 2:** Clinical characteristics, management and outcomes of thyroid eye disease patients. CAS, Clinical Activity Score; IOP, intraocular pressure; EOM, extraocular muscle; IV, intravenous; BD, twice daily; DON, dysthyroid optic neuropathy.

Patient No.	Age/Sex	Thyroid Status	Key Presentation	CAS (Activity)	IOP Status	Treatment Given	Outcome
1	20/F	Goiter present Hyperthyroidism	Lid edema, proptosis	3/7	24 & 26 mmHg	E/D Carboxymethylcellulose 1% 6t/d E/O hydroxypropyl methylcellulose HS E/D Brimonidine + timolol BD	Observation
2	34/M	Hyperthyroidism, smoker H/o ATT	Proptosis, pain and diminished vision, EOM restriction, diplopia, DON absent.	4/7	32 &28 mmHg	IV methylprednisolone pulse therapy for three days, one week followed by oral steroids (1gm/kg) for 12 weeks + Tab acetazolamide 250 mg TDS+ E/D Brimonidine+ Timolol BD.	Vision improved; persistent motility restriction
3	58/M	Hyperthyroidism	Proptosis, diplopia, DON absent.	3/7	35 &29 mmHg	IV methylprednisolone 500mg weekly for six weeks followed by 250mg IV for 6 weeks + Tab acetazolamide 250 mg BD+ E/D Brimonidine+ Timolol BD	Vision improved; residual deviation
4	42/M	Hyperthyroidism	EOM restriction, proptosis, diplopia, DON present.	4/7	40 & 35 mmHg	IV methylprednisolone pulse therapy 1gm for 3 consecutive days, weekly repeated 2 times followed by oral 1gm/kg for 8-12 weeks tapering doses+ Tab Acetazolamide 250mg TDS+ E/D Travoprost HS + Brinzolamide and Timolol combination.	Reduced proptosis; persistent restriction
5	25/F	Hyperthyroidism	Unilateral upper lid retraction.	1/7	18mmHg OU	E/D Carboxymethylcellulose 1% 6t/d E/O hydroxypropyl methylcellulose HS	Regular follow up

Clinical improvement, including reduction in inflammatory signs and orbital pain, was observed following treatment in our cases. However, persistent restrictive extraocular motility deficits were noted in two patients despite resolution of active inflammation. Elevated IOP was controlled with medical therapy in three of our patients, whereas in cases 3 and 4, IOP remained high on three months outcome and needed further lowering by adding AGMs. Also, none progressed to DON during follow-up.

Following intravenous methylprednisolone, visual acuity improved in affected patients, Proptosis reduced, and lid edema showed significant improvement. However, residual ocular deviation persisted, and extraocular motility restriction showed minimal improvement.

Patients were maintained on regular follow-up. Persistent restrictive myopathy suggested progression to the fibrotic stage in some cases. The follow-up duration ranged from 1 to 3 months. Patients with mild disease (cases 1 and 5) remained clinically stable on conservative therapy with no progression of disease during scheduled three-monthly follow-up visits. Patients with active moderate-to-severe TED (cases 2-4) required closer follow-up during intravenous methylprednisolone therapy. Patients with mild inactive disease managed conservatively were reviewed every three months, whereas those with active moderate-to-severe TED who received intravenous methylprednisolone pulse therapy were reviewed weekly during treatment, followed by monthly visits for the first three months and every three months thereafter. Patients with elevated IOP underwent more frequent monitoring until satisfactory IOP control was achieved. At each follow-up visit, BCVA, CAS, IOP, EOM, proptosis measurement, diplopia charting and anterior and posterior segment findings and need for additional treatment were documented (Table 3).

**Table 3:** Follow-up parameters of TED patients at three months. BCVA, Best corrected visual acuity; CAS, clinical activity score; IOP, intraocular pressure; GAT, Goldman applanation tonometer; AGMs, anti-glaucoma medications.

Case	BCVA		CAS		IOP (GAT)		Proptosis	EOM/ Diplopia	Additional treatment
	Baseline	Three months	Baseline	Three months	Baseline	Three months			
1	6/6 OU	6/6 OU	3/7	2/10	24 OD 26 OS	19 OD 20 OS	↓	Persistent restriction of EOM/ Diplopia absent	Anti-glaucoma medications (AGMs)
2	6/36 OD 6/24 OS	6/18 OD 6/9 OS	4/7	2/10	32 OD 28 OS	24 OD 22 OS	↓	Restricted movement and Diplopia persisted	Oral steroids for 12 weeks + AGMSs
3	6/36 OD 6/24 OS	6/9 OU	3/7	2/10	35 OD 29 OS	28 OD 22 OS	↓	Restricted movement persisted with diplopia	Oral steroid for 12 weeks + AGMs
4	6/18 OD 6/9 OS	6/9 OD 6/6 OS	4/7	2/10	40 OD 35 OS	29 OD 24 OS	↓	Persisted restricted EOM with diplopia	Oral steroids for 12 weeks + Low dose radiotherapy+ AGMs.
5	6/6 OU	6/6 OU	1/7	1/10	18 OU	19 OU	Not present	Not present	Not needed.

## Discussion

TED is a complex autoimmune disorder with a variable clinical course. Despite its relatively low incidence, the burden of disease is amplified by its chronicity and potential for disfigurement. Most patients develop mild disease; however, a subset progresses to moderate-to-severe or sight-threatening forms [3,6].

TED has a broad spectrum of clinical manifestations depending on disease activity and severity. Eyelid retraction is the most common presenting sign and results from fibrosis and overaction of the levator palpebrae superioris and Müller muscle. Proptosis occurs due to enlargement of extraocular muscles and expansion of orbital fat, producing increased orbital volume within the confined bony orbit. Restrictive extraocular myopathy, predominantly involving the inferior and medial rectus muscles, leads to diplopia and limitation of ocular motility.

Exposure keratopathy develops secondary to lagophthalmos and proptosis, predisposing patients to ocular surface disease. In severe cases, orbital apex crowding may compress the optic nerve, resulting in DON, which constitutes the most vision-threatening complication of TED.

Elevated IOP may occur because of increased episcleral venous pressure, mechanical compression of the globe by enlarged extraocular muscles, and impaired aqueous outflow, particularly in up gaze [3,6].

In our cases, proptosis was the most frequent clinical finding, followed by extraocular muscle restriction, diplopia, eyelid retraction, elevated IOP, and visual impairment due to active inflammatory disease. The majority of patients responded favorably to intravenous methylprednisolone therapy, although residual motility restriction persisted in patients presenting with advanced extraocular muscle fibrosis. Although the diagnosis of TED is usually straightforward in patients with established thyroid dysfunction and characteristic ocular findings, several orbital disorders may closely mimic its presentation.

Differential diagnoses include idiopathic orbital inflammatory disease (orbital pseudotumor), orbital lymphoma, cavernous hemangioma, lacrimal gland tumors, metastatic orbital tumors, carotid-cavernous fistula, orbital cellulitis, sarcoidosis, granulomatosis with polyangiitis, and congenital or acquired craniofacial asymmetry.

Unilateral proptosis, rapid progression, severe pain, atypical imaging findings, or absence of thyroid dysfunction should prompt further evaluation with orbital imaging and appropriate systemic investigations to exclude alternative diagnoses. In our series, a higher proportion of patients presented with advanced disease, including impending optic neuropathy. This may reflect delayed presentation or referral patterns, which are common in developing healthcare settings. Hereby, we have provided the algorithm for the initial workup of suspected TED patients for clinical guidance (Figure 7).

**Figure 7:**
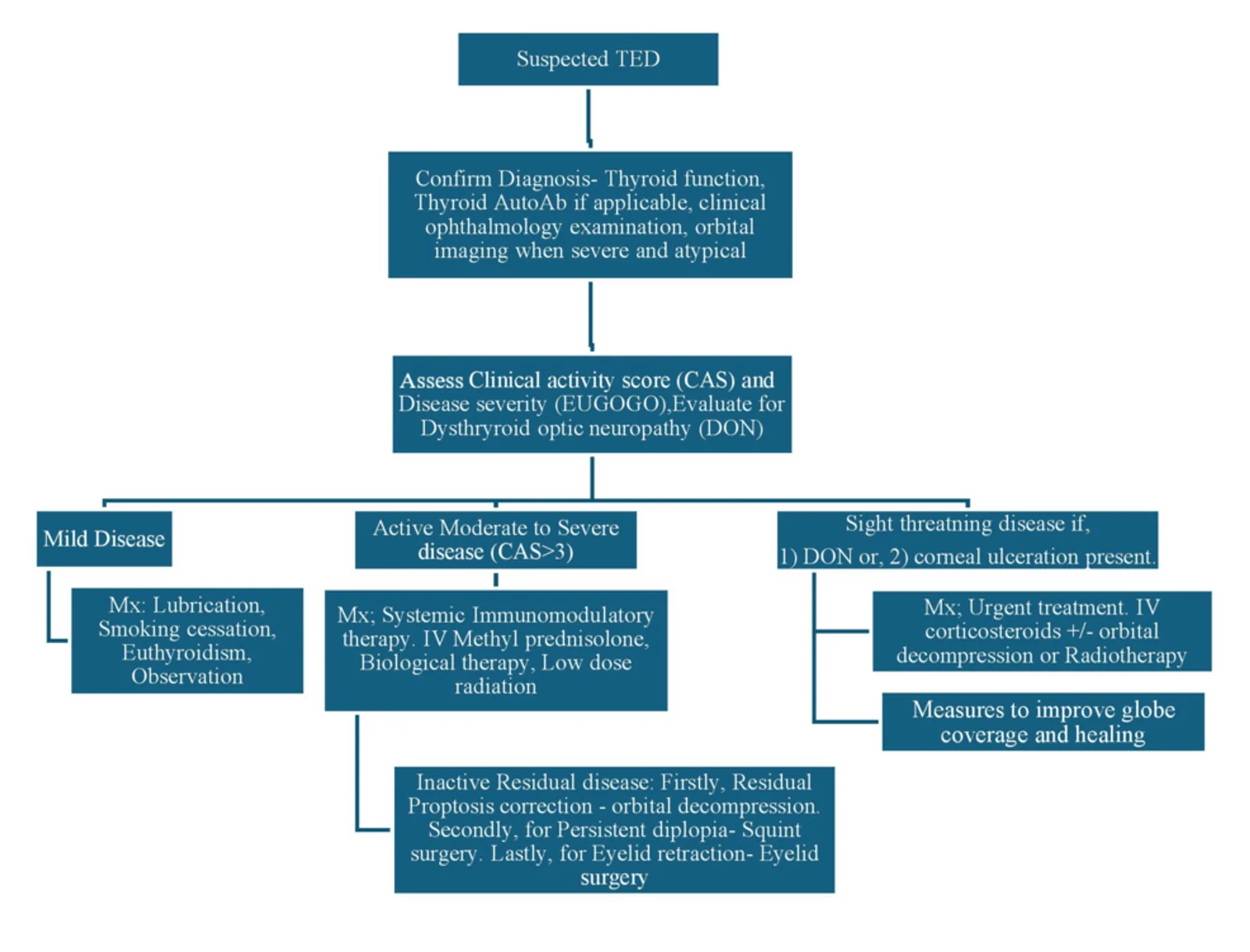
Flowchart diagram for initial evaluation of suspected TED for clinical guidance. CAS, Clinical Activity Score; EUGOGO, European group on Graves' Orbitopathy; DON, dysthyroid optic neuropathy; TED, thyroid eye disease

It is important to clearly distinguish between management of TED in active moderate-to-severe disease and sight-threatening disease. Intravenous corticosteroids are recommended as a systemic immunosuppressive treatment for active moderate-to-severe TED, whereas sight-threatening TED, particularly DON, requires urgent high-dose intravenous corticosteroid therapy and close monitoring of visual function as a mainstay of treatment. In our case series, intravenous methylprednisolone was used primarily for active inflammatory and restrictive disease; its use should not be interpreted as evidence that all treated patients had DON.

The main mechanisms of corticosteroids include inhibition of glycosaminoglycan deposition in orbital tissues, decrease of cytokine-mediated orbital edema, and quick control of the inflammatory cascade.

For active moderate-to-severe TED, like in our case 3, EUGOGO recommends intravenous methylprednisolone at a cumulative dose of 4.5 g administered as 500 mg weekly for six weeks followed by 250 mg weekly for 6 weeks; a higher cumulative dose of 7.5 g may be considered in selected patients with more severe disease.

Sight-threatening disease, particularly DON, requires urgent high-dose intravenous methylprednisolone (500-1000 mg daily for three consecutive or alternate days), with orbital decompression considered if the response is inadequate [7].

Another regimen of high-dose pulsed steroid therapy used in our setup for cases 2 and 4 was methylprednisolone (1 g) infused intravenously over 120 min daily for three successive days. This course was repeated at intervals of one week, until ophthalmopathy showed great improvement (up to four times). After the series of intravenous high-dose methylprednisolone therapy was finished, oral prednisolone treatment was started with an initial dose of 1mg/kg body weight. It was then gradually tapered and withdrawn within 3-12 months [9].

When used in pulse regimens, intravenous methylprednisolone has continuously shown better safety and efficacy than oral corticosteroids. Intravenous treatment has been shown to have response rates of roughly 70-80% in randomized controlled studies and meta-analyses, which are considerably greater than those seen with oral corticosteroids [10-12].

Additionally, intravenous regimens are linked to fewer systemic side effects and lower cumulative steroid dosages. Higher-dose regimens have been found to produce quick visual recovery in up to 80% of individuals with DON, frequently eliminating the need for immediate orbital decompression [13]. In line with previously reported results, individuals in the current case series who underwent intravenous methylprednisolone showed improvements in visual acuity, a decrease in proptosis, and the elimination of inflammatory symptoms including eyelid edema.

However, residual strabismus and chronic extraocular muscle restriction were seen, indicating the limited effectiveness of steroids after fibrotic remodeling stage of the disease had taken place. This observation is supported by studies indicating that while corticosteroids are highly effective during the active inflammatory phase, their impact on established fibrosis and restrictive myopathy is minimal [7,10]. Therefore, timing of intervention is critical, with early initiation offering the greatest potential for functional recovery. EUGOGO guidelines recommend that, when multiple procedures are required, orbital decompression should precede squint surgery and be followed by lid surgery as decompression changes the muscle alignment; squint surgery changes the lid position. Although surgery is not needed in all cases, it is done when disease becomes inactive and clinically stable with the exception of orbital decompression [7].

Overall, intravenous corticosteroids play a pivotal role in halting disease progression, preserving vision, and reducing the need for surgical intervention in selected cases. However, careful patient selection, adherence to recommended dosing protocols, and close monitoring for adverse effects-including hepatotoxicity and cardiovascular complications-remain essential. Raised IOP, observed in our four patients, may result from increased episcleral venous pressure due to orbital congestion or mechanical orbital compression by enlarged extraocular muscles. IOP may also be higher in up gaze and therefore should be assessed in relevant gaze positions when clinically indicated. In our patients, aqueous suppressants such as timolol and brimonidine were used to reduce aqueous production and control elevated IOP. Prostaglandin analogues increase uveoscleral outflow and may also be considered when additional IOP reduction is required; however, their use should be individualized in patients with active ocular surface inflammation because of the potential for conjunctival hyperemia and ocular surface intolerance. The choice of therapy depends on degree of IOP elevation, disease activity, ocular surface status, systemic contraindications and response to treatment. This requires vigilant monitoring and management.

The limitations of our study were the small sample size, retrospective design, short-term follow-up, and non-uniform availability of certain objective outcome measures. Importantly, the psychosocial burden of TED is significant. Facial disfigurement and diplopia adversely affect quality of life, reinforcing the need for a multidisciplinary approach involving ophthalmologists, endocrinologists, and oculoplastic surgeons.

## Conclusions

This case series emphasizes the heterogeneous clinical presentation of TED, the importance of early diagnosis, individualized management according to disease activity and severity, multidisciplinary care, and the need for long-term follow-up because restrictive ophthalmopathy may persist despite improvement in inflammatory activity.
